# Face masks effectively limit the probability of SARS-CoV-2 transmission

**DOI:** 10.1126/science.abg6296

**Published:** 2021-05-20

**Authors:** Yafang Cheng, Nan Ma, Christian Witt, Steffen Rapp, Philipp S. Wild, Meinrat O. Andreae, Ulrich Pöschl, Hang Su

**Affiliations:** 1 Max Planck Institute for Chemistry, 55128 Mainz, Germany.; 2 Institute for Environmental and Climate Research, Jinan University, Guangzhou 511443, China.; 3 Department of Outpatient Pneumology and Institute of Physiology, Charité Universitätsmedizin Berlin, Campus Charité Mitte, 10117 Berlin, Germany.; 4 University Medical Center of the Johannes Gutenberg-University Mainz, 55131 Mainz, Germany.; 5 Scripps Institution of Oceanography, University of California San Diego, La Jolla, CA 92093, USA.; 6 Department of Geology and Geophysics, King Saud University, 11451 Riyadh, Saudi Arabia.; 7 State Environmental Protection Key Laboratory of Formation and Prevention of Urban Air Pollution Complex, Shanghai Academy of Environmental Sciences, Shanghai 200233, China.

## Abstract

Airborne transmission by droplets and aerosols is important for the spread of viruses. Face masks are a well-established preventive measure, but their effectiveness for mitigating severe acute respiratory syndrome coronavirus 2 (SARS-CoV-2) transmission is still under debate. We show that variations in mask efficacy can be explained by different regimes of virus abundance and are related to population-average infection probability and reproduction number. For SARS-CoV-2, the viral load of infectious individuals can vary by orders of magnitude. We find that most environments and contacts are under conditions of low virus abundance (virus-limited), where surgical masks are effective at preventing virus spread. More-advanced masks and other protective equipment are required in potentially virus-rich indoor environments, including medical centers and hospitals. Masks are particularly effective in combination with other preventive measures like ventilation and distancing.

## Masking out air sharing

The effectiveness of masks in preventing the transmission of severe acute respiratory syndrome coronavirus 2 has been debated since the beginning of the COVID-19 pandemic. One important question is whether masks are effective despite the forceful expulsion of respiratory matter during coughing and sneezing. Cheng *et al.* convincingly show that most people live in conditions in which the airborne virus load is low. The probability of infection changes nonlinearly with the amount of respiratory matter to which a person is exposed. If most people in the wider community wear even simple surgical masks, then the probability of an encounter with a virus particle is even further limited. In indoor settings, it is impossible to avoid breathing in air that someone else has exhaled, and in hospital situations where the virus concentration is the highest, even the best-performing masks used without other protective gear such as hazmat suits will not provide adequate protection.

*Science*, abg6296, this issue p. 1439

## Teaser

Face masks are highly effective at preventing SARS-CoV-2 transmission because, in most environments, airborne virus abundance is limited.

Airborne transmission is one of the main pathways for the transmission of respiratory viruses, including the severe acute respiratory syndrome coronavirus 2 (SARS-CoV-2) (*1*). Wearing face masks has been widely advocated to mitigate transmission. Masks are thought to protect people in two ways: (i) source control, reducing the emission and spread of respiratory viruses through airborne droplets and aerosols, and (ii) wearer protection, reducing the inhalation of airborne respiratory viruses.

The effectiveness of masks, however, is still under debate. Compared with N95 or FFP2 respirators, which have very low particle penetration rates (~5%), surgical and similar masks exhibit higher and more variable penetration rates (~30 to 70%) (*2*, *3*). Given the large number of particles emitted upon respiration and especially upon sneezing or coughing (*4*), the number of respiratory particles that may penetrate masks is substantial, which is one of the main reasons for doubts about their efficacy in preventing infections. Moreover, randomized clinical trials have shown inconsistent or inconclusive results, with some studies reporting only a marginal benefit or no effect of mask use (*5*, *6*). Thus, surgical and similar masks are often considered to be ineffective. On the other hand, observational data show that regions or facilities with a higher percentage of the population wearing masks have better control of COVID-19 (*7*–*9*). So how are we to explain these contrasting results and apparent inconsistencies?

In this work, we develop a quantitative model of airborne virus exposure that can explain these contrasting results and provide a basis for quantifying the efficacy of face masks. We show that mask efficacy strongly depends on airborne virus abundance. On the basis of direct measurements of SARS-CoV-2 in air samples and population-level infection probabilities, we find that the virus abundance in most environments is sufficiently low for masks to be effective in reducing airborne transmission.

When evaluating the effectiveness of masks, we want to understand and quantify their effect on the infection probability, *P*_inf_. Assuming that every inhaled single virus (virion) has the same chance to infect a person, *P*_inf_ can be calculated by a single-hit model of infection

$$P_{\inf}=1-{\left(1-P_{\mathrm{single}}\right)}^{N_{v}}$$ (1)

where *P*_single_ represents the infection probability for a single virus and *N*_v_ represents the total number of viruses to which the person is exposed (*10*). For airborne transmission, the infection probability *P*_inf_ for a given time period can be plotted as a function of inhaled virus number, *N*_v_.

**Fig. 1:**
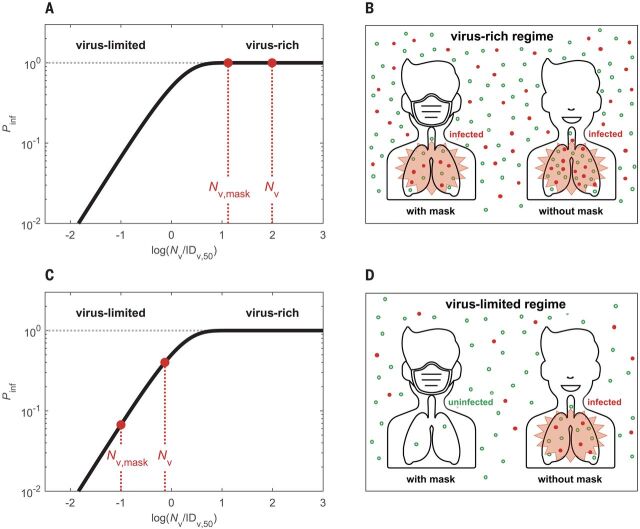
Schematic illustration of different regimes of abundance of respiratory particles and viruses. (**A** to **D**) The solid curves represent the infection probability (*P*_inf_) as a function of inhaled virus number (*N*_v_) scaled by median infectious dose ID_v,50_ at which *P*_inf_ = 50%. In the virus-rich regime [(A) and (B)], the concentration of airborne viruses is so high that both the numbers of viruses inhaled with and without masks (*N*_v,mask_, *N*_v_) are much higher than ID_v,50_, and *P*_inf_ remains close to ~1 even if masks are used. In the virus-limited regime [(C) and (D)], *N*_v_ and *N*_v,mask_ are close to or lower than ID_v,50_, and *P*_inf_ decreases substantially when masks are used, even if the masks cannot prevent the inhalation of all respiratory particles. In (B) and (D), the red dots represent respiratory particles containing viruses, and the open green circles represent respiratory particles without viruses. Man icon used in (B) and (D) was made by Tinu CA from www.freeicons.io, distributed under CC-BY 3.0.

Figure 1 illustrates the dependence of *P*_inf_ on *N*_v_ based on the single-hit model (Eq. 1) and scaled by the median infectious dose ID_v,50_ at which the probability of infection is 50% (*10*). It shows a highly nonlinear sensitivity of *P*_inf_ to changes in *N*_v_. Accordingly, the same percentage of change of *N*_v_ may lead to different changes in *P*_inf_ depending on the absolute level of *N*_v_. In a virus-rich regime, where *N*_v_ is much higher than ID_v,50_ (Fig. 1, A and B), *P*_inf_ is close to unity and is not sensitive to changes in *N*_v_. In this case, wearing a mask may not suffice to prevent infection. In a virus-limited regime, where *N*_v_ is close to or lower than ID_v,50_, however, *P*_inf_ strongly varies with *N*_v_, and reducing *N*_v_ by wearing a mask will lead to a substantial reduction in the infection probability (Fig. 1, C and D). Thus, we need to determine the regime of airborne virus abundance to understand mask efficacy.

Respiratory particles, including aerosol particles and larger droplets, can carry viruses and are often used to visualize the transmission of airborne viruses (*4*). Taking a representative average of respiratory activity (*11*), we find that a person typically emits a total number of ~3 × 10^6^ particles during a 30-min period (supplementary text, section S1.1). This very large number implies that indoor environments are usually in a respiratory particle–rich regime. Surgical masks with particle collection efficiencies of ~50% cannot prevent the release of millions of particles per person and their inhalation by others (see green dots in Fig. 1, B and D). In other words, the human-emitted respiratory particle number is so high that we cannot avoid inhaling particles generated by another person, even when wearing a surgical mask. If every respiratory particle were to contain one or more viruses, indoor environments would often be in a virus-rich regime because the median infectious dose ID_v,50_ for respiratory diseases is typically on the order of a few tens to thousands of viruses (*12*–*14*).

**Fig. 2:**
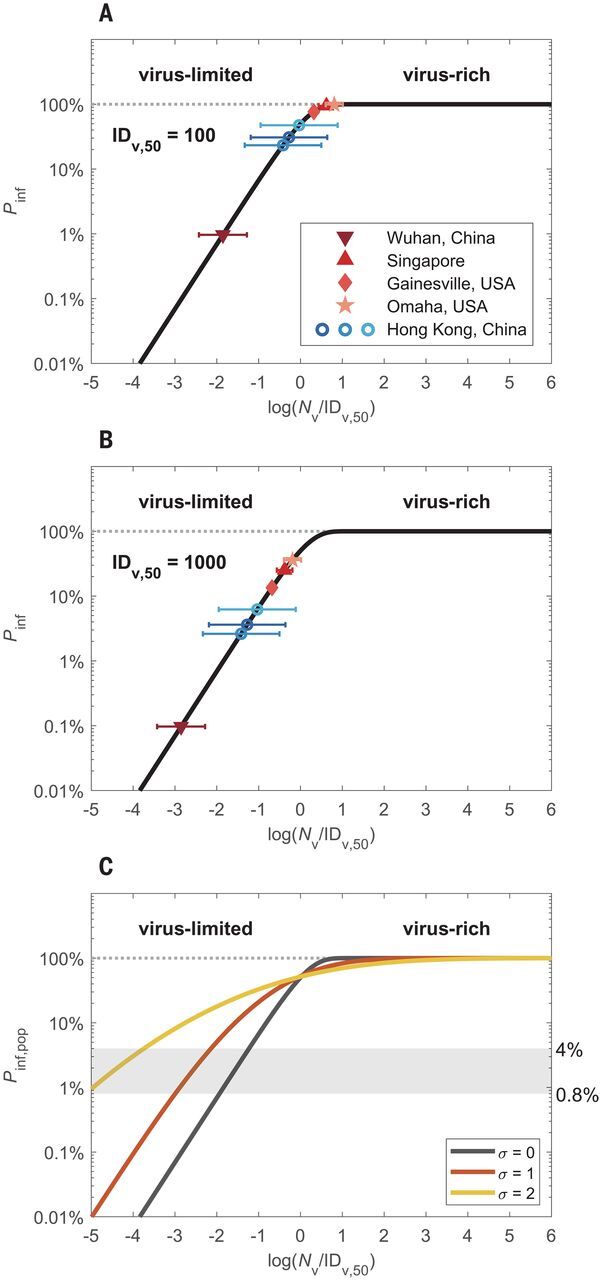
Infection probabilities and abundance regimes of SARS-CoV-2 and other respiratory viruses. (**A** and **B**) Individual infection probabilities (*P*_inf_) plotted against inhaled virus number (*N*_v_) scaled by characteristic median infectious doses of ID_v,50_ = 100 or 1000 viruses, respectively. The colored data points represent the mean numbers of viruses inhaled during a 30-min period in different medical centers in China, Singapore, and the US, according to measurement data of exhaled coronavirus, influenza virus, and rhinovirus numbers (blue circles) (*11*) and of airborne SARS-CoV-2 number concentrations (red symbols) (*15*–*18*), respectively. The error bars represent one geometric standard deviation. (**C**) Population-average infection probability (*P*_inf,pop_) curves assuming lognormal distributions of *N*_v_ with different standard deviations of σ = 0, 1, and 2, respectively. The *x* axis represents the mean value of log(*N*_v_/ID_v,50_). The shaded area indicates the level of basic population-average infection probability, *P*_inf,pop,0_, for SARS-CoV-2, as calculated from the basic reproduction number for COVID-19 and estimated values of average duration of infectiousness and daily number of contacts.

But, does a respiratory particle–rich regime actually imply a respiratory virus–rich regime? To answer this question, we investigated characteristic virus distributions in both exhaled air samples and indoor air samples including coronaviruses (HCoV-NL63, -OC43, -229E, and -HKU1), influenza viruses (A and B), rhinoviruses, and SARS-CoV-2 (supplementary text, section S1). We find that usually just a minor fraction of exhaled respiratory particles contains viruses. In contrast to the high number of emitted respiratory particles, the number of viruses in 30-min samples of exhaled air (*N*_v,30,ex_) are typically low, with mean values of ~53 for coronaviruses (HCoV-NL63, -OC43, -229E, and -HKU1), ~38 for influenza viruses (A and B), and ~96 for rhinoviruses (*11*) (supplementary text, section S1.2, and Fig. 2). Figure 2, A and B, shows the infection probabilities obtained by inserting the number of exhaled viruses (*N*_v,30,ex_) for the number of potentially inhaled viruses (*N*_v,30_), assuming a characteristic infectious dose of ID_v,50_ = 100 or 1000 viruses, respectively (*12*–*14*). For SARS-CoV-2 in various medical centers, we obtained mean values of *N*_v,30_ in the range of ~1 to ~600 (*15*–*18*) (supplementary text, section S1.3), which correspond to *P*_inf_ values in the range of ~0.1% to 10% for ID_v,50_ = 1000 and ~1% to 100% for ID_v,50_ = 100. The wide range of *N*_v,30_/ ID_v,50_ and *P*_inf_ values demonstrate that both virus-limited and virus-rich conditions can occur in indoor environments.

The high variabilities of *N*_v,30_ and *P*_inf_ shown in Fig. 2, A and B, are consistent with the wide distribution of viral load observed in respiratory tract fluids (*19*) and need to be considered for estimating population-average infection probabilities, *P*_inf,pop_ (supplementary text, section S4). For this purpose, we modeled *N*_v_ for SARS-CoV-2 as lognormally distributed with standard deviations (σ) in the range of ~1 to 2 on the basis of recently reported distributions of the viral load of SARS-CoV-2 in respiratory fluids (*19*) (supplementary text, section S4). As shown in Fig. 2C, the population-average infection probabilities with σ > 0 are higher than in the case of uniform exposure (σ = 0) in the virus-limited regime at *P*_inf,pop_ < ~50%. In other words, when the population-average infection probability is in the virus-limited regime with *P*_inf,pop,0_ < 0.5 (Fig. 2C), a broader distribution (larger σ) implies an increase in the fraction of transmission events under virus-rich conditions (e.g., superspreader events), which leads to a reduction of overall mask efficacy.

The basic reproduction number for COVID-19 (*R*_0_ ≈ 2 to 4) (*20*) can be related to a basic population-average infection probability, *P*_inf,pop,0_, through *R*_0_ = *P*_inf,pop,0_ × *c* × *d* (*21*). With the average duration of infectiousness (*d* ≈ 10 days) and average daily numbers of human contacts (*c* ≈ 10 to 25 contacts per day) (*22*, *23*), we obtain estimates in the range of ~0.8% to ~4% for *P*_inf,pop,0_, as indicated by the shaded area in Fig. 2C. The low levels of *P*_inf,pop,0_ indicate a widespread prevalence of virus-limited conditions.

**Fig. 3:**
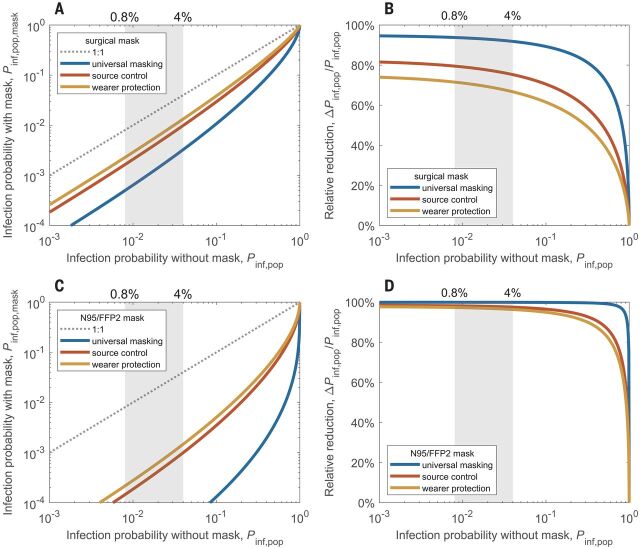
Reduction of airborne transmission by face masks worn by infectious persons only (source control), by susceptible persons only (wearer protection), or by all persons (universal masking). (**A** and **B**) Population-average infection probability in case of mask use (*P*_inf,pop,mask_) plotted against infection probability without face masks (*P*_inf,pop_) (A) and corresponding mask efficacy—i.e., relative reduction of infection probability, Δ*P*_inf,pop_/*P*_inf,pop_—plotted against *P*_inf,pop_ for surgical masks (B). (**C** and **D**) Same as (A) and (B) but for N95 or FFP2 masks; plots with linear scaling are shown in fig. S8. The lines represent the results obtained for source control (red line), wearer protection (yellow line), and the combination of both measures, i.e., universal masking, (blue line) in a population where the virus exposure is lognormally distributed with a standard deviation of σ = 1 (supplementary text, section S5). The shaded areas indicate the level of basic population-average infection probability, *P*_inf,pop,0_, corresponding to the basic reproduction number for COVID-19.

Different regimes of abundance are relevant not only for the distinction of respiratory particles and viruses, but also for different types of viruses. For example, viruses with higher transmissibility—i.e., those with higher loads and rates of emission and exhalation, greater environmental persistence, or lower ID_v,50_—may result in a virus-rich regime and lead to higher basic reproduction numbers, as observed for measles and other highly infectious diseases. Our analysis shows that the levels of *P*_inf_ and *R*_0_ can vary widely for different viruses. This means that aerosol transmission does not necessarily lead to a measles-like high *R*_0_ and that relatively low values of *P*_inf_ and *R*_0_ do not rule out airborne transmission. On the basis of the scaling with ID_v,50_, the curves shown in Figs. 1 to 3 can easily be applied to assess the efficacy of masks and other preventive measures against new and more-infectious mutants of SARS-CoV-2, such as B.1.1.7.

Figure 3 illustrates how the efficacies of surgical masks and N95 or FFP2 masks vary between virus-limited and virus-rich conditions when masks are worn only by infectious persons (source control), only by susceptible persons (wearer protection), or by all persons (universal masking). In Fig. 3A, the population-average infection probability in the case of surgical mask use (*P*_inf,pop,mask_) is plotted against the infection probability without masks (*P*_inf,pop_). It shows that surgical masking achieves large reductions in infection probability when the maskless infection probability is low but increasingly smaller reductions when the maskless infection probability is high. Figure 3B shows the corresponding mask efficacy, i.e., the percentage reduction of infection probability [Δ*P*_inf,pop_/*P*_inf,pop_ = (*P*_inf,pop_ − *P*_inf,pop,mask_)/ *P*_inf,pop_]. It decreases slowly with increasing *P*_inf,pop_ in the virus-limited regime, exhibits a steep decrease upon transition into the virus-rich regime as *P*_inf,pop_ approaches unity, and goes to zero at *P*_inf,pop_ = 1. Figure 3, C and D, shows equivalent plots for N95 or FFP2 masks.

**Fig. 4:**
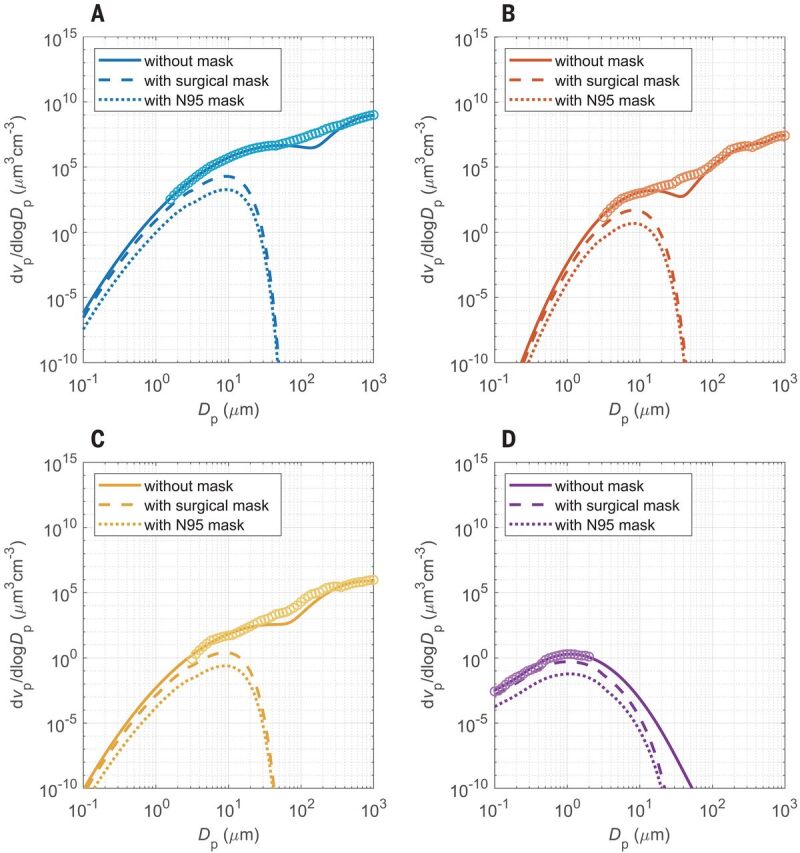
Volume size distributions of respiratory particles emitted during different respiratory activities with and without masks. (**A** to **D**) Distributions for sneezing (A), coughing (B), speaking (C), and breathing (D). The open circles are measurement data obtained without masks, and the solid lines are bi- or trimodal fits to the measurement data (*25*–*27*) (supplementary text, section S1.1). The dashed and dotted lines were obtained by scaling with the filter efficiency curves of surgical masks and of N95 or FFP2 masks, respectively (supplementary text, section S3). The symbols *v*_p_ and *D*_p_ represent the volume concentration and diameter of respiratory particles, respectively, and d*v*_p_/dlog *D*_p_ represents the volume distribution function (supplementary text, section S1.1).

Figure 3 illustrates that source control alone is more effective than wearer protection alone but that universal masking is the most effective. This is because masks are more effective in removing larger particles (Fig. 4), and freshly generated respiratory particles are usually largest at the source, shrinking upon evaporation in indoor air (*20*). Figure 3 accounts only for airborne transmission of viruses. When considering other forms of transmission, the relative importance of source control can be even higher (supplementary text, section S5) (*20*).

The nonlinear dependence of mask efficacy on infection risk differs from the assumption that the percentage change of infection probability as a result of mask use would be proportional to the percentage change of inhaled particle number (*20*). Under this assumption, wearing a mask would have the same effect on the transmission of a virus disease at any level of infection probability. Our analysis, however, shows that the efficacy of face masks depends strongly on the level of infection probability and virus abundance: Masks reduce the infection probability by as much as their filter efficiency for respiratory particles in the virus-limited regime but much less in the virus-rich regime (Fig. 3). Accordingly, experimental investigations may find low mask efficacies when they are performed under virus-rich conditions. Together with other influencing factors, like consistent and correct mask use (supplementary text, section S7.3), changes between virus-rich and virus-limited conditions may contribute to divergent results reported from laboratory studies and randomized controlled trials in different environments (*20*) (supplementary text, section S8). Notably, the increasing effectiveness of mask use at low virus abundance implies synergistic effects of combining masks with other preventive measures that reduce the airborne-virus concentration, such as ventilation and social distancing. For example, ventilation can change an environment from virus-rich to virus-limited conditions, which may be particularly important for medical centers with relatively high SARS-CoV-2 abundances (Fig. 2 and supplementary text, section S6). On the other hand, not only the efficacy of face masks but also the efficacy of distancing may be reduced in virus-rich environments (supplementary text, section S6). The more measures that are used, the more effective each measure will be in containing the virus transmission. If the inhaled dose may also affect the severity of infections (*14*), as is currently being debated (*24*), masks may still be useful even if the reduced dose still leads to an infection.

Figure 4 shows the size distribution of respiratory particles emitted by different human activities (*25*–*27*). Aerosols are physically defined as airborne solid or liquid particles with diameters smaller than 100 μm, which can remain suspended over extended periods of time. In medical studies, however, a threshold diameter of 5 μm has often been used to distinguish between a so-called aerosol mode and a so-called droplet mode. Our analysis of measurement data from exhaled and ambient air samples indicates that the so-called aerosol mode (<5 μm) contains more viruses than the so-called droplet mode (>5 μm) (*11*), although the latter comprises a larger volume of liquid emitted from the respiratory tract (tables S1 and S2). This may be explained by the following mechanisms: a higher viral load occurring in the lower respiratory tract where the smaller aerosol particles are generated (*28*) or an enrichment of organic surfactants and viruses upon the generation of smaller aerosol particles (*29*). Enrichment of viruses in the aerosol mode can enhance their transmission because smaller particles remain suspended for a longer time, which leads to stronger accumulation and dispersion in the air. This may cause higher airborne virus concentrations, inhaled virus numbers, and infection risks, especially in densely occupied rooms with poor ventilation and long periods of exposure. Moreover, small aerosol particles have a higher penetration rate and higher probability of reaching the lower respiratory tract (figs. S5 and S6).

Our analysis was focused on respiratory particles and droplets with diameters smaller than 100 μm [traditional physical definition of aerosols (*30*)]. Because of rapid gravitational settling, respiratory droplets larger than 100 μm are removed from the air in seconds, but they may still reach the upper respiratory tract of persons in close contact and cause infections by carrying large numbers of viruses in their very large liquid volume. For example, a single 1-mm droplet may carry as many as ~50,000 viruses in the case of a viral load of 10^8^ per milliliter of respiratory fluid, which is realistic and higher than the estimated infectious dose for SARS-CoV-2 (*14*). Such large droplets, however, are very efficiently (~100%) removed even by simple masks (Fig. 4 and supplementary text, section S3), which further emphasizes the importance and efficacy of face masks for preventing infections. Because of the strong size dependence, and to avoid ambiguities, we suggest that diameter range should be explicitly specified when discussing airborne transmission by fine respiratory aerosol particles or larger droplets.

Our results have important implications for understanding and communicating preventive measures against the transmission of airborne viruses, including SARS-CoV-2. When people see images or videos of millions of respiratory particles exhaled by talking or coughing, they may be afraid that simple masks with limited filtration efficiency (e.g., 30 to 70%) cannot really protect them from inhaling these particles. However, as only few respiratory particles contain viruses and most environments are in a virus-limited regime, wearing masks can keep the number of inhaled viruses in a low-*P*_inf_ regime and can explain the observed efficacy of face masks in preventing the spread of COVID-19. However, unfavorable conditions and the large variability of viral loads may lead to a virus-rich regime in certain indoor environments, such as medical centers treating COVID-19 patients. In such environments, high-efficiency masks and additional protective measures like efficient ventilation should be used to keep the infection risk low. The nonlinear dependence of mask efficacy on airborne virus concentration—i.e., the higher mask efficacy at lower virus abundance—also highlights the importance of combining masks with other preventive measures. Effective ventilation and social distancing will reduce ambient virus concentrations and increase the effectiveness of face masks in containing the virus transmission. Moreover, high compliance and correct use of masks is important to ensure the effectiveness of universal masking in reducing the reproduction number for COVID-19 (supplementary text, section S7.3, and fig. S11) (*20*).

## Supplementary Materials

science.sciencemag.org/content/372/6549/1439/suppl/DC1
Supplementary Text Figs. S1 to S14 Tables S1 to S6 References (*32*–*60*) MDAR Reproducibility Checklist
